# SARS-CoV-2 hijacks host CD55, CD59 and factor H to impair antibody-dependent complement-mediated lysis

**DOI:** 10.1080/22221751.2024.2417868

**Published:** 2024-10-22

**Authors:** Laura Gebetsberger, Zahra Malekshahi, Aron Teutsch, Gabor Tajti, Frédéric Fontaine, Nara Marella, André Mueller, Lena Prantl, Hannes Stockinger, Heribert Stoiber, Anna Ohradanova-Repic

**Affiliations:** a Medical University of Vienna, Center for Pathophysiology, Infectiology and Immunology, Institute for Hygiene and Applied Immunology, Vienna, Austria; b Medical University of Innsbruck, Institute of Virology, Innsbruck, Austria; c CeMM – Research Center for Molecular Medicine of the Austrian Academy of Sciences, Vienna, Austria

**Keywords:** SARS-CoV-2, complement, regulators of complement activation, immune evasion, antiviral immunity

## Abstract

The complement system is a vital anti-microbial defence mechanism against circulating pathogens. Excessive complement activation can have deleterious outcomes for the host and is consequently tightly modulated by a set of membrane-associated and fluid-phase regulators of complement activation (RCAs). Here, we demonstrate that severe acute respiratory syndrome coronavirus 2 (SARS-CoV-2) hijacks host cellular RCA members CD55 and CD59 and serum-derived Factor H (FH) to resist antibody-dependent complement-mediated lysis triggered by immunized human sera. Blockage of the biological functions of virion-associated CD55 and CD59 and competition of FH recruitment with functionally inactive recombinant FH-derived short consensus repeats SCR18-20 restore SARS-CoV-2 complement sensitivity in a synergistic manner. Moreover, complement-mediated virolysis is dependent on classical pathway activation and does not occur in the absence of virus-specific antibodies. Altogether, our findings present an intriguing immune escape mechanism that provides novel insights into the immunopathology observed in severe coronavirus disease 2019 (COVID-19).

## GRAPHICAL ABSTRACT

**Figure:**
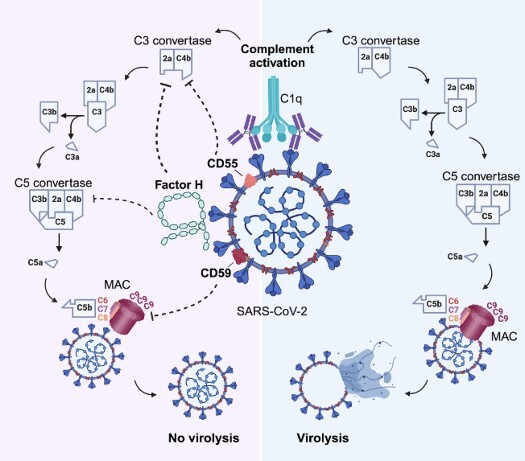

## Introduction

The complement system is an integral first-line defence mechanism against invading pathogens and comprises > 30 proteins in plasma, on cell surfaces or within host cells [1]. Complement operates in three pathways with distinct patterns of activation, the classical (CP), lectin (LP) and alternative pathways (AP), and results in three major outcomes: (i) opsonization and phagocytosis, (ii) chemotaxis of inflammatory immune cells and (iii) direct pathogen lysis [1,2]. The CP is initiated when the complement component C1q interacts with IgM or IgG immune complexes, or pathogen-associated molecular patterns on pathogen surfaces, leading to the activation of the multimeric C1 complex (C1qCr_2_Cs_2_) [2]. Recognition of carbohydrate motifs on non-self surfaces by mannose binding lectin (MBL), collectins and ficolins activates the LP, and the AP is fuelled by the constant low-grade hydrolysis (“tick-over”) of C3 to C3(H_2_O), or by C3b via the amplification loop (AL) [1–3]. All three pathways converge on the formation of a C3 convertase, which cleaves the central complement component C3. This generates C3b, which promotes pathogen opsonization and phagocytosis and further drives the formation of the C5 convertases, which cleave C5 and thereby activate the terminal pathway. In this, the generated C5b sequentially associates with C6, C7, C8 and multiple C9 molecules and assembles the membrane attack complex (MAC, C5b-9), a small pore that triggers pathogen lysis by disrupting the integrity of cell membranes or viral envelopes [1,2]. The cleavage of C3 and C5 further produces the anaphylatoxins C3a and C5a, which initiate potent inflammatory responses through the chemotaxis of immune cells via their cognate receptors C3aR, C5aR and C5L2 [2].

To prevent bystander damage to host cells, complement is tightly regulated by both fluid-phase and membrane-associated proteins (“regulators of complement activation,” RCAs) [3]. These include the plasma membrane-resident protectin (CD59) and decay accelerating factor (DAF, CD55) as well as the fluid-phase regulator Factor H (FH), which shield self surfaces from complement-mediated injury through (i) inhibiting MAC formation (CD59), (ii) destabilizing CP/LP and/or AP C3 convertases (“decay acceleration,” CD55, FH), and (iii) exerting cofactor activity for Factor I-mediated C3b inactivation (FH) [3,4]. Importantly, numerous pathogens have subverted these regulatory mechanisms through various strategies, including (i) the hijacking of host RCAs, (ii) enzymatic inactivation of host complement factors, and (iii) the production of complement-like proteins which mimic RCA functions [5,6].

Severe acute respiratory syndrome coronavirus 2 (SARS-CoV-2), the aetiological agent of coronavirus disease 2019 (COVID-19), is a large enveloped positive-sense single-stranded RNA virus in the *Coronaviridae* family [7]. The viral genome encodes four structural proteins, the core nucleocapsid (N) and surface spike (S), envelope (E) and membrane (M) proteins, as well as 16 non-structural (nsp) and 9 accessory ORF proteins, which assist viral replication [7,8]. Clinical manifestations of SARS-CoV-2 infection are highly variable, ranging from mild upper respiratory tract symptoms in most patients to severe disease characterized by an uncontrolled state of hyperinflammation, acute respiratory distress syndrome (ARDS), multiorgan failure and death [9,10]. Complement activation has been heavily implicated in the pathogenesis of severe COVID-19, reflected by elevated levels of C3a, C5a, and soluble C5b-9 (sC5b-9) in the plasma of critically ill patients, as well as the deposition of activated complement products in injured organs [10–14]. The crosstalk between complement and coagulation has been further associated with the COVID-19-mediated coagulopathy and thromboinflammation [11,15,16].

Despite the extensive research on the role of complement in COVID-19, not much is known about the interaction of SARS-CoV-2 with RCAs. Here, we demonstrate that SARS-CoV-2 hijacks host cellular CD55 and CD59 as well as serum-derived FH to resist antibody-dependent complement-mediated lysis triggered by immunized human sera. Blockage of the biological functions of virion-incorporated CD55 and CD59 and inhibition of FH recruitment restore the complement sensitivity of SARS-CoV-2 in a synergistic manner. Moreover, complement-mediated virolysis seems to depend on CP activation and is not observed in the absence of virus-specific antibodies. Altogether, we present an intriguing immune escape mechanism, which may contribute to the complement-driven pathology observed in severe COVID-19.

## Materials and methods

Materials and methods are provided in the supplemental data.

## Results

### Association of host cell-derived CD55 and CD59 with purified SARS-CoV-2 particles

**Figure 1.**
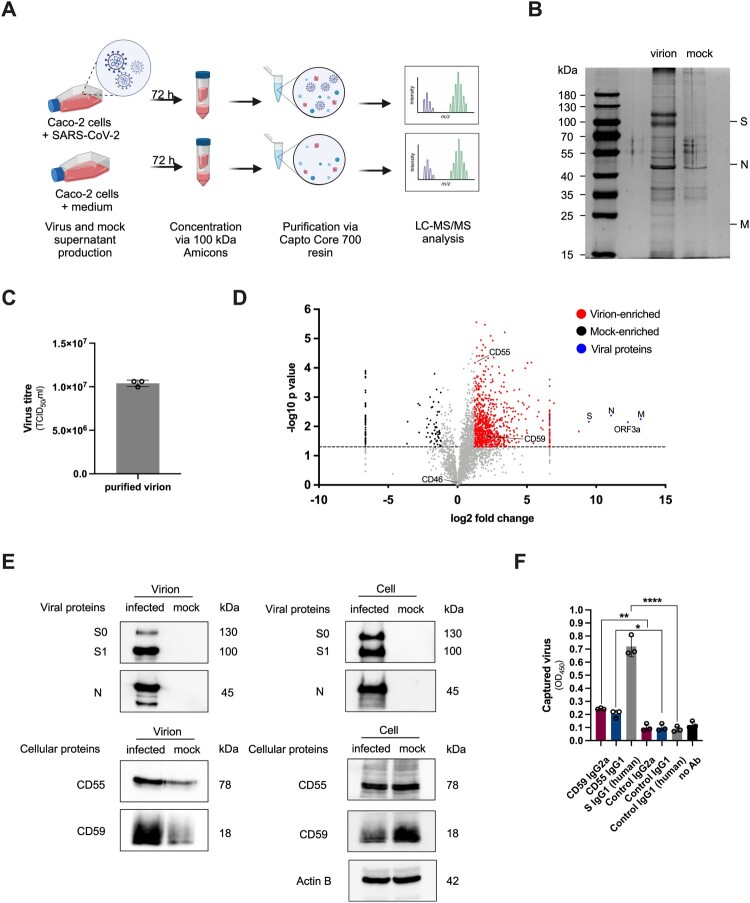
Association of host cell-derived CD55 and CD59 with purified SARS-CoV-2 particles. (A) Experimental setup for SARS-CoV-2 and mock purifications. Virus-containing and mock supernatants from Caco-2 cells were harvested 72 hpi, concentrated, and purified by size exclusion using Capto Core 700 resin. Purified virus and mock preparations were lysed and analysed by LC-MS/MS. Created with Biorender.com. (B) Reducing 10% SDS-PAGE and silver staining analysis of purified SARS-CoV-2 and mock preparations. The positions of structural viral proteins, identified by their predicted molecular weights, are indicated. (C) Viral titres of purified SARS-CoV-2, determined by TCID_50_ assay. Data are represented as mean ± SD (*n* = 3 independent experiments). (D) Scatter plot of enriched viral (blue dots) and host cellular proteins in SARS-CoV-2 (red dots) and mock (black dots) preparations (*p* < 0.05, *log2 fold change* ≥ 1.2). Non-enriched proteins are depicted in grey, and the horizontal line indicates p = 0.05. Statistical significance of 3 independent experiments was evaluated by two-tailed Student’s *t*-test. (E) Immunoblot analysis of viral uncleaved S0, cleaved S1 and N proteins and host cellular proteins associated with SARS-CoV-2 and mock preparations (left) and Caco-2 cell lysates (right). (F) Detection of virion-associated CD55 and CD59 and viral S protein by virus capture ELISA. Data are represented as mean ± SD (*n* = 3 independent experiments). Statistical significance was assessed by one-way ANOVA with Tukey’s multiple comparisons test; *p *<* 0.05, **p < 0.01, ****p < 0.0001.

The incorporation of host cellular proteins into budding virions of enveloped viruses is a well-described phenomenon [17–20]. To detect host cellular complement factors associated with SARS-CoV-2, we analysed the protein content of purified cell culture-derived virus particles by liquid chromatography and tandem mass spectrometry (LC-MS/MS). Briefly, SARS-CoV-2-containing and mock (conditioned cell culture medium) supernatants were produced in the naturally permissive Caco-2 cells, followed by purification using a size exclusion-based methodology, described elsewhere [21] (Figure 1A). The purity and integrity of virus and mock preparations were routinely verified by silver staining and Western blotting, which validated that uncleaved (S0) and cleaved (S1) viral S and N proteins were exclusively detected in the purified virus supernatants (Figure 1B and Figure 1E). Moreover, median tissue culture infectious dose (TCID_50_) assay revealed a virus titre of ∼10^7^ TCID_50_/ml (Figure 1C). Purified virus and mock samples were subsequently characterized by LC-MS/MS analysis, which yielded a total of 816 virus-enriched proteins that were reproducibly identified in 3 biological replicates with a log2 fold change of ≥1.2 (*p*-value <0.05) (Figure 1D, Table S1). The structural viral S, N and M proteins were highly enriched in the isolated virions compared to the purified mock supernatants (Figure 1D, blue dots). Due to its small size (8 kDa), the E protein likely generated only a few tryptic peptides and therefore was not detected. Instead, we observed high amounts of the accessory protein ORF3a, which was described as a virion-associated factor for the phylogenetically related SARS-CoV [22]. Other viral accessory proteins, including ORF6, ORF7a, ORF9b and polyprotein 1a (pp1a) were also enriched in the virus fraction, although to a lower magnitude than the structural proteins or ORF3a (Table S1). Apart from viral proteins, LC-MS/MS analysis further unveiled 808 enriched host cellular proteins in our purified virus preparations (Figure 1D, red dots), 40 of which were exclusively detected in the isolated virions (Table S1). To decipher putative functions of the virion-incorporated cellular proteins, we performed gene enrichment analyses using the online DAVID tool. KEGG Pathway and GO Biological Process analyses revealed an enrichment of ribosomal, proteasomal and COVID-19 disease-related proteins, as well as proteins involved in translation, and various biosynthetic and metabolic processes, respectively (Figure S1A-B). GO Molecular Function profiling further highlighted an abundance of RNA binding, heterocyclic compound and protein binding properties (Figure S1C). This is in line with a recently published analysis of SARS-CoV-2 particles budding from two lung epithelial cell lines [23], suggesting that SARS-CoV-2 associates with a conserved set of host cellular proteins.

Importantly, we consistently detected the complement regulators CD55 and CD59 among the virion-enriched proteins in all three preparations from infected Caco-2 cells (Figure 1D, Table S1). To discriminate between proteomes of virus particles and extracellular vesicles such as exosomes, which due to similar size and shared biogenesis pathways are often copurified with virions, we designated proteins as virion-specific only when they displayed a higher relative abundance in the virus-enriched fraction compared to the mock fraction. Accordingly, CD46, another RCA member which is commonly identified in virus preparations, was also detected in our LC-MS/MS analysis but due to the lack of statistical enrichment in the isolated virions was excluded from any functional assays (Figure S1D, Table S1). The absence of the classical exosome markers CD81, CD63 and TSG101 in the virus fraction (Table S1) further validated minimal exosome contamination [23].

The association of host cell-derived CD55 and CD59 with SARS-CoV-2 particles was verified by Western blotting (Figure 1E), and plate-based capture ELISA (Figure 1F), which allowed us to precipitate the virions using plate-bound CD55, CD59 and S monoclonal antibodies (mAbs), followed by lysis and quantification of the viral N protein. The detection of N as a readout further facilitates the discrimination between viruses and non-viral vesicles, assuming that viral proteins are not present in the latter. CD59 and CD55 mAbs captured on average 33.76% and 27.99% of the S-captured virus, respectively, and significantly higher amounts than the corresponding isotype control mAbs (Figure 1F). Since the used mAb clones (MEM-43 for CD59, and BRIC-216 for CD55) recognize functional surface epitopes [24,25], we conclude that CD59 and CD55 decorate the virion surface presumably in a functional state.

### Virion-associated CD55 and CD59 confer resistance to Ab-dependent complement-mediated lysis (ADCML)

**Figure 2.**
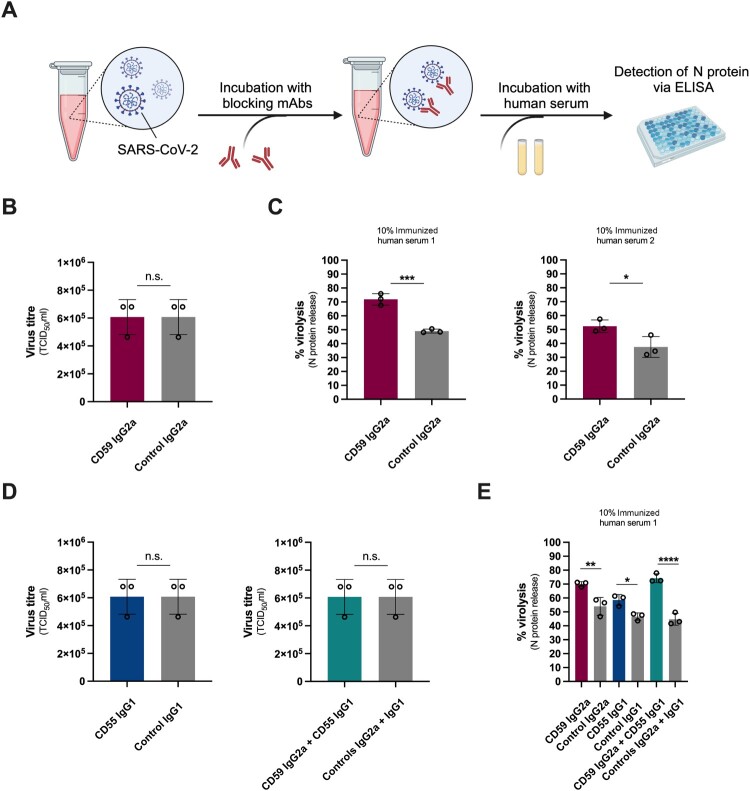
Virion-associated CD55 and CD59 confer resistance to ADCML. (A) Experimental setup for the analysis of ADCML of SARS-CoV-2 pre-treated with CD59 and/or CD55 blocking mAbs. Created with BioRender.com. (B, D) Titres of SARS-CoV-2 incubated with CD59 (B) and/or CD55 blocking mAbs (D), or isotype control mAbs, determined by TCID_50_ assay. Data are represented as mean ± SD (*n* = 3 independent experiments) and statistical significance was evaluated by two-tailed unpaired Student’s *t*-test. (C, E) Enhancement of ADCML of SARS-CoV-2 by CD59 (C), and/or CD55 blocking mAbs (E), and isotype control mAbs. Virolysis is expressed as % of detergent Triton X-100-treated virions. Data are represented as mean ± SD (*n* = 3 independent experiments) and statistical significance was assessed by two-tailed unpaired Student’s *t*-test; *p < 0.05, **p < 0.01, ***p < 0.001, ****p < 0.0001.

The acquisition of functional CD55 and CD59 by enveloped viruses has been described as a clever strategy to resist complement-mediated lysis [18,26,27]. To determine whether the incorporated CD55 and CD59 confer any protective effects to SARS-CoV-2, we tested the stability of the virus particles against human complement. Accordingly, normalized inputs of SARS-CoV-2 were pre-treated with blocking mAbs against CD59, CD55 or both, followed by the incubation with immunized normal human sera (iNHS) from healthy donors as a source of complement (Figure 2A). SARS-CoV-2 S- and receptor binding domain (RBD)-specific IgG Abs were detectable in all iNHS (Figure S2A), as well as neutralizing Abs against the SARS-CoV-2 ancestral strain (Figure S2B). Human sera were used at a concentration of 10%, which is representative of complement levels on mucosal surfaces [27]. Complement-mediated virolysis was determined by quantifying the released viral N protein by ELISA (Figure 2A) using heat-inactivated (HI) sera- or cell culture medium-treated virions and detergent Triton X-100-treated virions as negative and maximum lysis controls, respectively.

Treatment of SARS-CoV-2 with CD59 and/or CD55 blocking mAbs did not affect infectivity, determined by TCID_50_ assay (Figure 2B and Figure 2D). However, as is shown in Figure 2C (grey bars), SARS-CoV-2 is partially resistant to ADCML by two iNHS, when compared to Triton X-100-treated virions. Blockage of the biological function of virion-associated CD59 significantly enhances virolysis (Figure 2C, pink bars), implying a protective role of the hijacked RCA member. The maximum lysis capacities of the sera positively correlate with the levels of S- and RBD-specific IgGs, as well as neutralizing Ab titres (Figure 2C and Figure S2A-B). Blockage of virion-incorporated CD55 also boosts ADCML, and the inhibition of both RCAs exhibits the most dramatic effect (Figure 2E). Targeting CD14, a non-RCA host cellular protein identified in the virion-enriched fraction (Figure S1D, Table S1) or the use of a CD59 non-blocking mAb (MEM-43/5) [24] did not affect complement-mediated virolysis (Figure S3A-B), indicating that the enhancement of lysis is specific to the inhibition of RCA members and the blockage of their functional sites. Moreover, the presence of EDTA, which suppresses the activation of all three complement pathways through Ca^2+^ and Mg^2+^ chelation [28] completely abrogates the lytic activity of iNHS (Figure S3C), verifying that the observed lysis is exclusively driven by complement.

To exclude any confounding activation of human complement via the Fc parts of the used murine CD55 and CD59 blocking mAbs, we furthermore generated a Fab fragment of the CD59 blocking mAb MEM-43 by papain digestion, along with a control Fab recognizing an unrelated protein that was not detected in our LC-MS/MS analyses. We verified the purity and specificity of the Fabs by silver staining (Figure S4A) and flow cytometry (Figure S4B, left column), respectively. Both Fabs also successfully competed with the corresponding full-length mAbs for binding to target cells (Figure S4B, right column), and incubation of SARS-CoV-2 with the CD59 Fab did not alter infectivity (Figure S4C). Importantly, the CD59 Fab significantly enhanced ADCML of SARS-CoV-2 compared to treatment with both the control Fab and isotype control mAb, and its blocking capacity rivalled that of the intact CD59 mAb (Figure S4D).

### PI-PLC treatment validates the role of CD55 and CD59 in SARS-CoV-2 complement resistance

**Figure 3.**
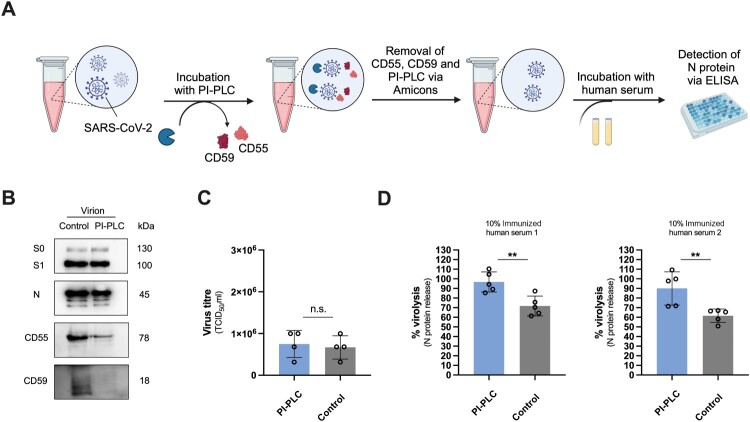
PI-PLC treatment validates the role of CD55 and CD59 in SARS-CoV-2 complement resistance. (A) Experimental setup for the analysis of ADCML of SARS-CoV-2 pre-treated with PI-PLC. Created with BioRender.com. (B) Immunoblot analysis of PI-PLC- or vehicle control-treated SARS-CoV-2 to verify CD55 and CD59 removal by enzymatic treatment. (C) Titres of PI-PLC- or vehicle control-treated SARS-CoV-2, determined TCID_50_ assay. Data are represented as mean ± SD (*n* = 4 independent experiments) and statistical significance was evaluated by two-tailed unpaired Student’s *t*-test. (D) ADCML of PI-PLC- or vehicle control-treated SARS-CoV-2, expressed as % of Triton X-100-treated virions. Data are represented as mean ± SD (*n* = 5 independent experiments) and statistical significance was assessed by two-tailed unpaired Student’s *t*-test; **p < 0.01.

To confirm our findings, we furthermore tested the complement sensitivity of SARS-CoV-2 pre-treated with phosphatidylinositol-specific phospholipase C (PI-PLC). PI-PLC cleaves glycosylphosphatidylinositol (GPI)-anchored proteins [18] and since both CD55 and CD59 are GPI-linked, we assumed that enzymatic treatment would remove them from the virion surface. Accordingly, SARS-CoV-2 particles were incubated with PI-PLC, followed by the removal of the cleaved GPI-anchored proteins and PI-PLC via Amicon ultracentrifugal units. The pre-treated virions were then exposed to iNHS and complement-mediated virolysis was determined by N protein ELISA (Figure 3A). PI-PLC treatment efficiently erased CD59 and partially removed CD55 from the virion surface but not the viral S protein (Figure 3B) and had no effect on viral titres (Figure 3C). Similarly to CD55 and CD59 blocking mAbs, PI-PLC treatment significantly increased complement-mediated lysis of SARS-CoV-2 compared to vehicle control-treated virus particles, for both iNHS (Figure 3D). Altogether, these results demonstrate that SARS-CoV-2 utilizes its host cell-acquired CD55 and CD59 to resist ADCML.

### Interaction of SARS-CoV-2 with FH and recombinant FH-derived SCR18-20

**Figure 4.**
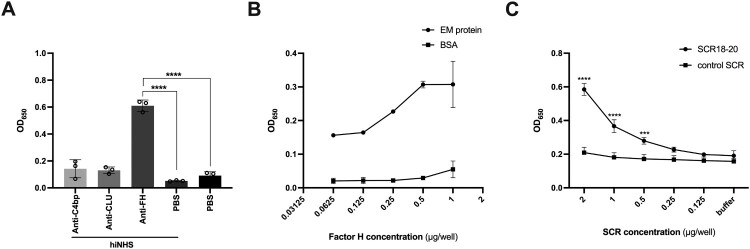
Interaction of SARS-CoV-2 with FH and recombinant FH-derived SCR18-20. (A) Binding of HI-niNHS-derived FH, C4bp and CLU to immobilized SARS-CoV-2 particles, assessed by ELISA. Data are represented as mean ± SD (*n* = 3 independent experiments) and statistical significance was evaluated by one-way ANOVA with Tukey’s multiple comparisons test; ****p < 0.0001. (B) Interaction of plate-bound His-tagged SARS-CoV-2 EM protein or BSA (control) with FH, determined by ELISA. Data are represented as mean ± SD (*n* = 2 independent experiments). (C) Interaction of recombinant FH-derived SCR18-20 and control SCR with immobilized SARS-CoV-2 particles, determined by ELISA. Data are represented as mean ± SD (*n* = 3 independent experiments), and statistical significance was assessed by two-way ANOVA with Sidak’s multiple comparisons test; ***p < 0.001, ****p < 0.0001.

Apart from cell-associated RCAs, complement is further regulated by a set of soluble proteins which are present in plasma [3]. Interestingly, several viruses and bacteria hijack soluble RCAs to attenuate complement activation on the microbial surface, thereby ensuring their survival [29–32]. To investigate the interaction of SARS-CoV-2 with fluid-phase RCAs, we incubated plate-bound virus particles with HI-NHS, followed by the detection of the CP/LP inhibitor C4b binding protein (C4bp), the AP regulator FH and the MAC inhibitor clusterin (CLU) by ELISA [3,33]. While we captured only negligible amounts of C4bp and CLU, SARS-CoV-2 potently bound serum-derived FH (Figure 4A). To elucidate which part of the virion surface mediates this interaction, we further assessed the binding of serum-derived FH to recombinant SARS-CoV-2 structural proteins (S and an E and M fusion protein (EM)). No interaction was observed between FH and the S protein, neither in its recombinant form nor with VSV pseudotyped with the SARS-CoV-2 S protein (data not shown). In contrast, the EM fusion protein adhered to FH in a concentration-dependent manner (Figure 4B).

FH is a 155 kDa glycoprotein composed of 20 individually folded, homologous short consensus repeat (SCR) domains [5]. While the N-terminal SCR1-4 harbour the complement regulatory properties, the C-terminal SCR19-20 contain the heparin-binding motifs essential for host cell attachment [5,33]. Numerous pathogens hijack FH via its SCR18-20 or SCR19-20 domains to achieve protection from complement [33]. Accordingly, recombinant FH-derived SCR18-20 or SCR19-20, which encompass the C-terminal self-recognition domains, but lack any regulatory function, can compete with full-length FH for pathogen binding, without exerting any protective effects. We have previously shown that recombinant SCR18-20 and SCR19-20 significantly enhance Ab-induced complement-dependent cytotoxicity of primary tumour cells, whereas no effects were observed for the non-heparin-binding control SCRs, which do not contain any known host cell-binding or complement regulatory domains [34–36]. To investigate SCR binding to SARS-CoV-2, we analysed the interaction of plate-bound virions with SCR18-20 and a control SCR by ELISA. Importantly, our results demonstrate that SCR18-20 potently binds to SARS-CoV-2 in a concentration-dependent manner, whereas no specific binding was observed for the control SCR (Figure 4C).

### SCR18-20 abrogates FH-mediated protection from complement-mediated virolysis

**Figure 5.**
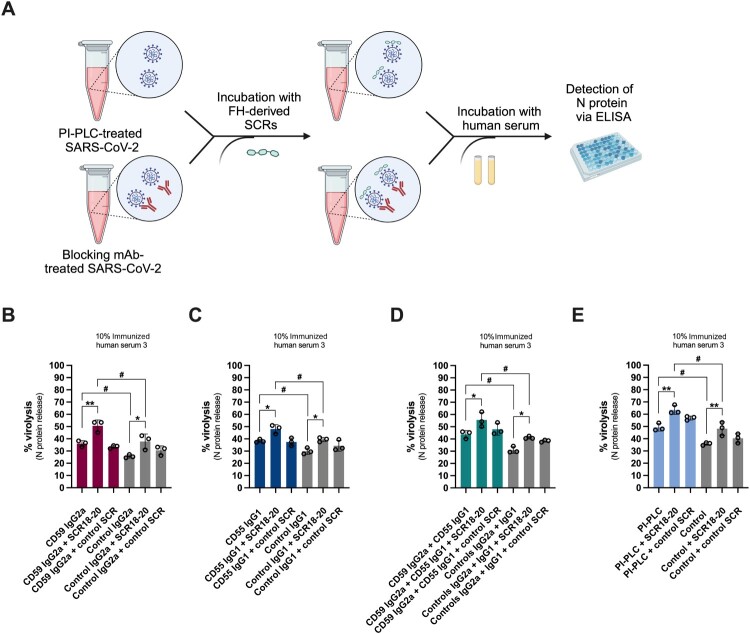
SCR18-20 abrogates FH-mediated protection from ADCML. (A) Experimental setup for the analysis of ADCML of CD59 and/or CD55 blocking mAb(s)- or PI-PLC-treated SARS-CoV-2 in the presence of recombinant FH-derived SCR18-20, or a control SCR. Created with BioRender.com. (B-E) Contribution of FH-derived SCR18-20 or control SCR to ADCML of SARS-CoV-2 treated with CD59 (B), CD55 (C) blocking mAb, or both (D), or PI-PLC (E). The effects of the SCRs alone are shown in grey. Virolysis is expressed as % of Triton X-100-treated virions. Data are represented as mean ± SD (*n* = 3 independent experiments), and statistical significance was evaluated by one-way ANOVA with Tukey’s multiple comparisons test; *p < 0.05, **p < 0.01 and two-tailed unpaired Student’s *t*-test; *#p* < 0.05.

To decipher whether FH recruitment contributes to SARS-CoV-2 complement resistance, we included recombinant FH-derived SCR18-20 in our serum sensitivity assay. Briefly, SARS-CoV-2 particles were either used unmodified (controls) or pre-treated with CD55 and/or CD59 blocking mAbs, or PI-PLC, as described above, followed by the incubation with iNHS in the presence of FH-derived SCR18-20, or a control SCR (Figure 5A). Importantly, the addition of SCR18-20 increases complement-mediated virolysis by an average of 20%, compared to control-treated (isotype control mAbs for CD55 and CD59, or vehicle control for PI-PLC) SARS-CoV-2 (Figure 5B–E, grey bars), thereby confirming a role of FH in complement resistance. The control SCR does not significantly alter virolysis in any of the conditions. Combining SCR18-20 with PI-PLC, or CD59 and/or CD55 blocking mAbs boosts virolysis even further (Figure 5B–E, coloured bars), indicating that FH acts synergistically with CD55 and CD59 on the virion surface. Thus, we conclude that SARS-CoV-2 collectively utilizes serum-derived FH and the incorporated CD55 and CD59 to resist ADCML.

### SARS-CoV-2 lysis depends on the presence of virus-specific Abs

**Figure 6.**
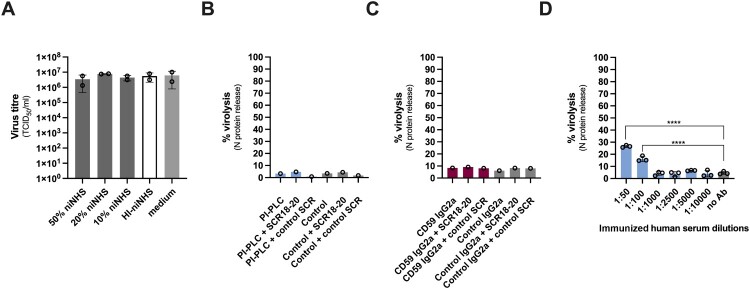
No lysis of SARS-CoV-2 in the absence of virus-specific antibodies. (A) Titres of SARS-CoV-2 incubated with niNHS, HI-niNHS, or cell culture medium in the indicated concentrations, determined by TCID_50_ assay. Data are represented as mean ± SD (*n* = 2 independent experiments). (B) niNHS-dependent complement-mediated lysis of SARS-CoV-2 treated with PI-PLC (blue bars) or vehicle control (grey bars), in the presence of FH-derived SCR18-20 or a control SCR. Virolysis is expressed as % of Triton X-100-treated virions. One biological replicate performed in duplicates is shown. (C) niNHS-dependent complement-mediated lysis of SARS-CoV-2 incubated with CD59 mAb (pink bars) or isotype control mAb (grey bars), in the presence of FH-derived SCR18-20 or a control SCR. Virolysis is expressed as % of Triton X-100-treated virions. One biological replicate performed in duplicates is shown. (D) Enhancement of niNHS-dependent complement-mediated lysis by SARS-CoV-2-specific pAbs. 10% niNHS was used as a source of complement and spiked with pAbs present in HI-iNHS in the indicated dilutions. Virolysis is expressed as % of Triton X-100-treated virions. Data are represented as mean ± SD (*n* = 3 independent experiments), and statistical significance to was evaluated by one-way ANOVA with Dunnett’s multiple comparisons test; ****p < 0.0001.

While the classical complement pathway is primarily triggered by pathogen-specific Abs, activation of LP and AP is Ab-independent [2]. To determine whether complement-mediated lysis occurs in the absence of virus-specific Abs, we incubated SARS-CoV-2 with non-immune NHS (niNHS) purchased before the start of the COVID-19 pandemic (Figure S2C), followed by virus titration via TCID_50_ assay. Interestingly, titres of niNHS-treated SARS-CoV-2 remained completely unchanged compared to HI-niNHS-, or medium only-treated virus particles, even when niNHS was used at a concentration of 50% (Figure 6A). This indicates that no detectable lysis has occurred. Similarly, quantification of the released N protein from niNHS-treated SARS-CoV-2 revealed no significant virolysis, regardless of the presence of SCR18-20, or pre-treatment with PI-PLC (Figure 6B) or CD59 blocking mAb (Figure 6C). Spiking niNHS with HI-iNHS, which rescues CP activation, increases virolysis in a concentration-dependent manner (Figure 6D), verifying that our niNHS does contain functional complement, but cannot trigger lysis in the absence of virus-specific Abs. Of note, several studies have demonstrated Ab-independent complement activation by SARS-CoV-2 *in vitro* [1,37,38]. Thus, it is plausible that the complement cascade is also initiated by our niNHS, resulting in opsonization of virus particles. However, the activation may be incomplete or insufficient, falling below the threshold required for MAC assembly and consequently virolysis.

### Discussion

Uncontrolled complement activation can have detrimental consequences and constitutes a core clinical feature of severe COVID-19 and post-COVID-19 sequelae, including multisystem inflammatory syndrome in children (MIS-C), a rare but potentially life-threating complication following SARS-CoV-2 infection, and Long COVID [10,11,39,40].

Here, we demonstrate that SARS-CoV-2 hijacks host-derived RCA members CD55, CD59 and FH to resist ADCML induced by complement-competent immunized human sera. Using LC-MS/MS analysis of purified virions, we unveil that CD55 and CD59 are incorporated into mature SARS-CoV-2 particles (Figure 1D–E) and decorate the virion surface in fully functional confirmations (Figure 1F), whereas FH is recruited from human serum via its SCR18-20 domains through the interaction with the viral E and M proteins (Figure 4A–C). Confronting SARS-CoV-2 with active complement revealed a partial resistance to complement-mediated lysis, which was significantly reduced by inhibiting the biological functions of virion-incorporated CD55 and CD59 either with specific blocking mAbs (Figure 2C and Figure 2E) and Fabs (Figure S4D), or cleavage by PI-PLC (Figure 3D) and/or preventing FH recruitment using recombinant FH-derived SCR18-20 (Figure 5B–E). In this respect, blockage of CD59 generated a higher degree of lysis than CD55 inhibition, possibly resulting from i) higher efficacy of CD59 in preventing complement-mediated lysis due to its direct MAC-inhibitory properties, ii) more efficient incorporation of CD59 into virus particles (Figure 1F), or iii) higher potency of the used CD59 blocking mAb. The combination of CD55 and CD59 mAbs or PI-PLC treatment with FH-derived SCR18-20 achieved the highest degree of lysis (Figure 5B–E), indicating that the hijacked RCAs act synergistically on the virion surface through their key roles in regulating different steps of the complement cascade. Importantly, the intrinsic lysis capacities of the used sera depend on the levels of SARS-CoV-2-specific Abs and correlate with both S- and RBD-specific IgGs and nAbs (Figure 2C and Figure S2A-B). In contrast, no virolysis was observed in the absence of virus-specific Abs, even when the protective functions of all contributing RCAs were inhibited (Figure 6), implying that CP activation may be required for virolytic activity *in vitro*.

All three complement pathways are activated during SARS-CoV-2 infection [1,11,41-43]. Ab-independent complement activity was mainly attributed to the LP [1,37,41], whereas canonical AP activation *in vitro* requires the presence of a cell surface and occurs through the interaction of the viral S protein with membrane-associated heparan sulphates [38]. In our experiments, we observed that the introduction of SARS-CoV-2-specific polyclonal antibodies (pAbs) readily induced virolysis (Figure 6D), and that this effect was further enhanced by blocking the protective effects of the AP regulator FH (Figure 5B–E). From these findings, we deduce that both CP activation and the interplay with the AL and/or LP contribute to virolysis. Nevertheless, the CP may play a critical role in driving the complement-related pathology in COVID-19, an inference that is supported by the documented association between early virus-specific IgM and/or IgG Ab responses and complement hyperactivation, leading to tissue injury in critically ill patients [12,43,44].

Evasion or delay of complement-mediated clearance through the acquisition of host RCAs can lead to several consequences. Firstly, resistance to MAC-mediated lysis likely generates complement-opsonized virus particles, which can interact with complement receptor (CR)-bearing cells. In particular, a recent study reported that C3c- and C3d-opsonized SARS-CoV-2 activates monocyte-derived dendritic cells in a CR3- and CR4-dependent manner and thereby induces potent type I interferon and inflammatory responses [37]. Although the CR-mediated cytokine response was abrogated by the presence of virus-specific Abs through FcγRII (CD32) engagement, CR3 and CR4 are also highly expressed on monocytes and monocyte-derived macrophages, two key players in the COVID-19-associated hyperinflammation, which may respond differently to Ab- and complement-opsonized virus particles [45,46]. Secondly, C3b/C4b opsonization of SARS-CoV-2 may promote the interaction with CR1-expressing erythrocytes. In this regard, Kisserli et al. [47] showed that a subset of critically ill COVID-19 patients exhibits substantial C4d deposition on erythrocytes, suggesting the handling of immune complexes and/or complement-coated virus particles. The observed C4d deposition was accompanied by a decrease in CR1 surface expression, which due to the inhibitory role of CR1, may promote further activation of the complement cascade and amplification of immune responses. In conclusion, both these and our findings emphasize the necessity for additional research to comprehensively uncover the immunological implications of the complement system in SARS-CoV-2 infection, with the potential to understand the immunopathology of severe COVID-19.

## Supplementary Material

Table_S1.xlsx

Rev_Gebetsberger_et_al_Emerg_Microbes_Infect_Supplemental_data_clean.docx
