# Supplementary material for: SARS-CoV-2 hijacks host CD55, CD59 and factor H to impair antibody-dependent complement-mediated lysis
Source: Emerg Microbes Infect. 2024 Oct 22;13(1):2417868. doi: 10.1080/22221751.2024.2417868 (PMC11520101; doi:10.1080/22221751.2024.2417868)
Supplement: Rev_Gebetsberger_et_al_Emerg_Microbes_Infect_Supplemental_data_clean.docx [file TEMI_A_2417868_SM7364.docx]

**Supplemental data**

**SARS-CoV-2 hijacks host CD55, CD59 and Factor H to impair antibody-dependent complement-mediated lysis**

Laura Gebetsberger, Zahra Malekshahi, Aron Teutsch, Gabor Tajti, Frédéric Fontaine, Nara Marella, André Mueller, Lena Prantl, Hannes Stockinger, Heribert Stoiber and Anna Ohradanova-Repic

## Methods

### Resource availability

Resources and reagents used in the study are detailed in Table S2. Further information and requests for resources and reagents should be directed to and will be fulfilled by the lead contact, Anna Ohradanova-Repic ([anna.repic@meduniwien.ac.at](mailto:anna.repic@meduniwien.ac.at)).

### Ethical statements

This study was performed in accordance with the Declaration of Helsinki and the research was approved by the Ethics Committee of the Medical University of Vienna (1238/2024).

### Cell lines and viruses

Caco-2 and Vero cells were cultured in Dulbecco’s Modified Eagle’s Medium (DMEM), high glucose, GlutaMAX, pyruvate (Gibco) supplemented with 100 U/ml penicillin, 100 µg/ml streptomycin (Gibco), 1% MEM non-essential amino acids solution (Gibco) and 10% foetal calf serum (FCS, Biowest) at 37°C and 5% CO_2_. Vero76 and Vero76-ACE2 cells were cultured in DMEM (Thermo Fisher Scientific) supplemented with 100 U/ml penicillin, 100 µg/ml streptomycin (Thermo Fisher Scientific), 2 mM L-glutamine (Thermo Fisher Scientific) and 10% FCS, and additionally with blasticidin and hygromycin B (InvivoGen) for Vero76-ACE2 cells to maintain the transfected ACE2 plasmid. Jurkat E6.1 cells were cultured in Roswell Park Memorial Institute (RPMI) 1640 medium supplemented with 100 U/ml penicillin, 100 µg/ml streptomycin, (Gibco), 2 mM L-glutamine (Gibco), 1% MEM non-essential amino acids solution (Gibco) and 10% FCS. Authentic SARS-CoV-2 isolate BetaCoV/Germany/BavPat1/2020 (ancestral strain) was kindly provided by Christian Drosten (Charité, Berlin, Germany) and distributed by the European Virology Archive (Ref-SKU: 026V-03883). SARS-CoV-2 isolate 1.2 (ancestral strain, isolated at the Medical University of Innsbruck, 2020) was kindly provided by the group of Janine Kimpel (Institute of Virology, Medical University of Innsbruck). For the generation of virus stocks, SARS-CoV-2 was propagated in Caco-2, Vero or Vero76 cells in complete DMEM with reduced serum (2% FCS) at 37°C and 5% CO_2_. Supernatants were harvested 48-72 hours post infection (hpi), centrifuged and/or filtered through 0.45 µM filters and stored in aliquots at -80°C. All experiments with infectious SARS-CoV-2 were performed under Biosafety Level 3 (BSL-3) containment.

### Human serum

Immunised normal human sera (iNHS) were obtained from healthy, SARS-CoV-2-vaccinated donors and stored in aliquots at -80°C. Non-immune normal human serum (niNHS) was purchased from Dunn Labortechnik GmbH (Asbach, Germany) and stored in aliquots at
-80°C. Heat-inactivation (HI) was performed at 56°C for 30 min.

### SARS-CoV-2 purification

Three T175 cm^2^ flasks with 80-90% confluent Caco-2 cells were infected with SARS-CoV-2 isolate BetaCoV/Germany/BavPat1/2020 (ancestral strain) at a multiplicity of infection (MOI) of 0.05 in serum-free DMEM, high glucose, GlutaMAX, pyruvate (#10569010, Gibco) supplemented with 100 U/ml penicillin, 100 µg/ml streptomycin (Gibco), 1% MEM non-essential amino acids solution (Gibco) and 5 mM HEPES pH 7.0-7.6 (Gibco), or mock-infected with equal volumes of medium. Virus-containing and mock supernatants were harvested 72 hpi, clarified from cell debris by centrifugation (2000 g, 15 min, 4°C) and concentrated to 1 ml, using Amicon ultracentrifugal filter units (100 kDa MWCO, Merck Millipore). Concentrated supernatants were subsequently purified using Capto Core 700 multimodal chromatography resin (Cytiva), as described [1]. Briefly, 1 ml of the concentrated samples was mixed with a 50% slurry of Capto Core 700 resin in Hank’s Balanced Salt Solution (HBSS, Gibco) and purified by six sequential extractions of end-over-end rotation for 10-15 min at room temperature (RT). Purified virus and mock preparations were stored at -80°C for further analyses.

### TCID_50_ assay

Virus titres were quantified by adding serial dilutions of the virus samples to Vero cells cultured in 2% FCS DMEM at 37°C and 5% CO_2_. The median tissue culture infectious dose (TCID_50_/ml) was determined by examining the cytopathic effect of the infected cells and calculated according to Reed and Muench or Spearman and Karber [2].

### Western blot

Purified virus and mock supernatants were lysed in 5x Laemmli buffer (250 mM Tris HCl pH 6.8, 10% SDS, 50% glycerol, 0.05% bromophenol blue) and boiled at 95°C for 10 min for virus inactivation. Caco-2 cell lysates were prepared in NP-40 buffer (150 mM NaCl, 50 mM Tris-HCl pH 8.0, 1% NP-40) supplemented with cOmplete™ Protease Inhibitor Cocktail (Roche) and mixed with 5x Laemmli. If required, lysates were additionally supplemented with 1% β-mercaptoethanol (Sigma) and boiled at 95°C for 5min to achieve reducing conditions. Samples were then separated by 10% SDS-PAGE and transferred to PVDF membranes (Immobilon®-P, Merck Millipore), followed by blocking with 5% dry skimmed milk in TBS+0.05% Tween (TBST). Proteins were visualised with primary antibodies (Abs) diluted in 3% BSA in TBST and appropriate horseradish peroxidase (HRP)-conjugated secondary Abs diluted in 5% dry skimmed milk in TBST, followed by chemiluminescent detection (ECL Prime Western Blot detection, Cytiva or Westar Hypernova, 7BioScience) via LAS 4000 (Fujifilm). Image analysis was performed using open-source image analysis software package Fiji.

**Primary Abs:** SARS-CoV-2 S1 monoclonal Ab (mAb; #40591-MM42, SinoBiological, 1:1000), SARS-CoV-2 N mAb (#40143-MM05, SinoBiological, 1:1000), CD55 mAb (#31759, Cell Signaling, 1:1000), CD59 mAb (#25393, Cell Signaling, 1:1000), actin polyclonal Ab (pAb; #A2066, Sigma, 1:1000), CD46 mAb (#13241, Cell Signaling, 1:1000), CD14 mAb (clone MEM-18, kindly provided by Vaclav Horejsi, Institute of Molecular Genetics, Academy of Sciences of the Czech Republic, Prague, Czech Republic, 1:1000).

**Secondary Abs:** anti-rabbit IgG-HRP conjugate (#1706515, BioRad, 1:3000), anti-mouse IgG-peroxidase Ab (#A9044, Sigma, 1:10000).

### Silver staining

Lysates of purified virus and mock supernatants in 5x Laemmli buffer were mixed with 1% β-mercaptoethanol (Sigma) and boiled at 95°C for 5 min to achieve reducing conditions. 10 µl of purified lysates and 1 µl of PageRuler™ Prestained Protein Ladder (Thermo Fisher Scientific) were loaded on 10% SDS gels. CD59 mAb MEM-43 (kindly provided by Vaclav Horejsi, Institute of Molecular Genetics, Academy of Sciences of the Czech Republic, Prague, Czech Republic), mouse IgG2a isotype control mAb (#400264, BioLegend), CD59 Fab MEM-43 and control Fab (CD48 MEM-102) were mixed with 2x Laemmli buffer and separated on a 10% SDS gel (500 ng/well) under non-reducing conditions. ExcelBand™ 3-color High Range Protein Marker (#PM2600, SMOBIO Technology) was used at 1 µl/well. Silver staining was performed as described [3]. Images were acquired with LAS 4000 (Fujifilm).

### Sample preparation for mass spectrometry analysis

Purified SARS-CoV-2 virus and mock samples were lysed in 2% SDS, 50 mM HEPES pH 7.0-7.6, 1 mM PMSF supplemented with Protease Inhibitor Cocktail (Sigma), boiled at 95°C for 10 min for virus inactivation and snap-frozen in liquid nitrogen. Inactivated samples were then processed using an adapted Single-Pot solid-phase-enhanced sample preparation (SP3) methodology [4]. Briefly, proteins were reduced by incubation with 10 mM DTT for 1 h at 56°C, cooled down to RT, followed by alkylation of the reduced cysteines with 55 mM iodoacetamide for 30 min in the dark. The reduced and alkylated samples were then incubated with 400 μg of an equal mixture of paramagnetic carboxylate modified beads (SpeedBeads 45152105050250 and 65152105050250, Cytiva, pre-washed with LC-MS grade water (LiChrosolv, Merck Millipore) and reconstituted at 50 µg/ml) for 5 min at RT, and the formed protein-bead complexes were precipitated with 70% acetonitrile. Particles were then immobilised using a magnetic rack (DynaMag-2 Magnet, Thermo Fisher Scientific), the supernatant was discarded, and SDS was removed by washing twice with 200 μl 70% ethanol and once with 180 μl 100% acetonitrile. Samples were then resuspended in 100 μl of 50 mM NH_4_HCO_3_ and digested by incubation with 1 μg of trypsin overnight at 37°C. After digestion, samples were acidified to a final concentration of 1% trifluoroacetic acid (TFA; Uvasol, Merck Millipore), desalted and concentrated using stage tips with two stacked C18 plugs (Empore, Merck Millipore) [5]. Stage tips were activated with three times 100 μl acetonitrile and equilibrated with three times 100 μl of 0.4% formic acid, 2% TFA in H_2_O before loading the samples. Salts were cleaned up with 100 μl of 0.1% TFA and peptides were eluted using two times 50 μl 90% acetonitrile, 0.4% formic acid, directly into MS vials. Finally, eluates were dried in a vacuum concentrator and reconstituted in 10 μl of 0.1% TFA.

### Liquid chromatography coupled to tandem mass spectrometry (LC-MS/MS)

Mass spectrometry was performed on an Orbitrap Fusion Lumos mass spectrometer (Thermo Fisher Scientific) coupled to an Dionex Ultimate 3000RSLC nano system (Thermo Fisher Scientific) via nanoflex source interface. Tryptic peptides were loaded onto a trap column (Pepmap 100 5 μm, 5 × 0.3 mm, Thermo Fisher Scientific) at a flow rate of 10 μL/min using 0.1% TFA as loading buffer. After loading, the trap column was switched in-line with a 50 cm, 75 µm inner diameter analytical column (packed in-house with ReproSil-Pur 120 C18-AQ, 3 μm, Dr. Maisch, Ammerbuch-Entringen, Germany). Mobile-phase A consisted of 0.4% formic acid in water and mobile-phase B of 0.4% formic acid in a mix of 90% acetonitrile and 10% water. The flow rate was set to 230 nL/min and a 90 min gradient used (4 to 24% solvent B within 82 min, 24 to 36% solvent B within 8 min and, 36 to 100% solvent B within 1 min, 100% solvent B for 6 min before bringing back solvent B at 4% within 1 min and equilibrating for 18 min). Analysis was performed in a data-dependent acquisition mode. Full MS scans were acquired with a mass range of 350 - 1650 m/z in the orbitrap at a resolution of 120,000 (at 200 m/z). Monoisotopic peak determination was set to peptides with inclusion of charge states between 2 and 5, automatic gain control (AGC) was set to a target of 2 × 10^5^, and a maximum injection time of 80 ms was applied. Precursor ions for MS2 analysis were selected using a TopN dependent scan approach with a cycle time of up to 20 scans. MS2 spectra were acquired in the orbitrap (FT) using a quadrupole isolation window of 1.6 Da and higher energy collision induced dissociation (HCD) at a fixed normalised collision energy (NCE) of 30%. The resolution was set to 15,000 (at 200 m/z) with a fixed first mass of 120 m/z, an AGC target set to 5 × 10^4^, and a maximum injection time set to 100 ms. Dynamic exclusion for selected ions was 30 s, and isotopes were excluded. A single lock mass at m/z 445.120024 [6] was employed. Xcalibur version 4.3.73.11 and Tune 3.4.3072.18 were used to operate the instrument.

### Mass spectrometry data analysis

Acquired raw data files were processed with Proteome Discoverer 2.4, using the database search engine Sequest HT. Searches were performed with full tryptic digestion against the reviewed Swiss-prot Human database containing isoforms (Version 052020, 42,289 sequences) as well as the reviewed Swiss-prot SARS-CoV-2 database (Version 022022, 17 sequences), with up to two miscleavage sites. Data were searched with mass tolerances of ± 10 ppm on the precursor level and 0.025 Da for fragmented ions. Oxidation (+15.9949 Da) of methionine was set as a variable modification, while carbamidomethylation (+57.0214 Da) of cysteine residues was set as a fixed modification. Percolator was used to curate for false positives, and results were further filtered for a false discovery rate of 1% at peptide and protein levels. Automated chromatographic alignment and feature mapping were enabled. MS1 quantification was performed using the Minora Feature Detector and the Precursor Ions Quantifier nodes. Protein abundances were calculated via the summed abundances of their unique peptides. Downstream data analysis was performed in R, for normalisation of each condition (total peptide abundances), statistical analysis, and data visualisation. The mass spectrometry data has been deposited to the ProteomeXchange Consortium via the PRIDE partner repository [7] with the dataset identifier PXD050009.

### Bioinformatic analyses

Gene Ontology and KEGG pathway analyses were performed using the online DAVID tool (<https://david.ncifcrf.gov>) [8,9]. GOTERM_BP_ALL, GOTERM_MF_ALL functional annotation and KEGG_PATHWAY annotations charts were selected, and the top 10 categories (with p value <0.05) were plotted with Excel365 (Microsoft).

### Preparation of Fab fragments

The Fab fragments were produced by standard papain digestion of the parental mAb in the presence of β-mercaptoethanol as described previously [10,11].

### PI-PLC treatment

Phosphatidylinositol-specific phospholipase C (PI-PLC, Sigma Aldrich) was reconstituted in 10 mM Tris-HCl pH 7.4, 144 mM NaCl and 0.05% BSA, aliquoted and stored at -80°C. For experiments, normalised inputs of SARS-CoV-2 were incubated with 5 U/ml PI-PLC solution, or equal volumes of reconstitution buffer (vehicle control) for 1 h at 37°C. The cleaved glycosylphosphatidylinositol (GPI)-anchored proteins and residual enzyme were removed by centrifugation, using Nanosep™ centrifugal filters (300 kDa MWCO, Thermo Fisher Scientific) and/or Amicon ultracentrifugal filter units (100 kDa MWCO, Merck Millipore). PI-PLC-treated SARS-CoV-2 was either lysed in 5x Laemmli buffer for Western Blot analysis or used for TCID_50_ and serum sensitivity assays.

### Virus capture ELISA

Nunc™ MaxiSorp™ ELISA plates (BioLegend) were coated with CD59 mAb MEM-43, CD55 mAb BRIC-216 (#sc-59092, Santa Cruz Biotechnology), SARS-CoV-2 S1 mAb AM001414 (#938701, BioLegend), and isotype control mAbs (#400264, #400101, #403501, all from BioLegend) at 10 µg/ml overnight at 4°C. Plates were washed three times with PBS, blocked with 10% FCS+3% BSA in PBS and incubated with 50 µl (corresponding to 1.41 x 10^5^ TCID_50_) of SARS-CoV-2 per well for 2 h at 37°C to allow virus capture. Plates were washed four times with PBS to remove unbound virus, followed by lysis of the plate-bound virus with 0.5% Triton X-100 and quantification of the released viral N protein by ELISA, as described below.

### SARS-CoV-2 N ELISA

Nunc™ MaxiSorp™ ELISA plates (BioLegend) were coated with SARS-CoV-2 N mAb (#940902, BioLegend) at 1 µg/ml overnight at 4°C. Plates were then washed three times with PBS and blocked with 10% FCS+3% BSA in PBS, followed by incubation with SARS-CoV-2 samples at 50 µl/well for 1 h at RT. After four washing steps with PBS, plates were fixed with 5% formalin (Sigma) in PBS for virus inactivation and incubated with rabbit anti-SARS-CoV-2 N mAb (#40143-R019, SinoBiological, 1:10000 in blocking buffer) and goat anti-rabbit IgG HRP-conjugated Abs (#1706515, BioRad, 1:5000 in blocking buffer). ELISA was developed using the DY999 substrate solution (R&D Systems) according to the manufacturer’s instructions and OD_450_ was measured with either a Mithras Multimode Plate Reader (Berthold Technologies), Tecan Spark® Multimode Microplate Reader (Tecan) or an Epoch Microplate Spectrophotometer (BioTek).

### Serum sensitivity ELISA

Normalised inputs of SARS-CoV-2 particles were incubated with CD59 blocking mAb MEM-43 and/or CD55 blocking mAb BRIC-216, CD14 blocking mAb MEM-18, CD59 non-blocking mAb MEM-43/5 (kindly provided by Vaclav Horejsi), or isotype control mAbs (#400264 and #400101 from BioLegend, mouse IgG1 isotype control mAb PPV06 and mouse IgG2b isotype control mAb PPV02, kindly provided by Vaclav Horejsi) at 30 µg/ml for 45 min at RT, or alternatively treated with PI-PLC as described above. CD59 Fab MEM-43 and control Fab (CD48 MEM-102) were used at 30 µg/ml for 45 min at RT. When indicated, recombinant Factor H (FH)-derived short consensus repeat SCR18-20, or a control SCR (SCR11-12) were added to the pre-treated virions at 50 µg/ml for 30 min at 37°C. To determine complement sensitivity, SARS-CoV-2 virions were then incubated with 10% complement-competent normal human serum (NHS), 10% heat-inactivated (HI)-NHS and cell culture medium (negative controls), or 1% Triton X-100 (maximum lysis control) for 1 h at 37°C. Virolysis was determined by quantifying the released viral N protein by ELISA as described above. Lysis is expressed as % released N protein of Triton X-100-treated virions and was calculated using the following formula: *[(OD_450_ of complement-competent NHS-treated SARS-CoV-2 – OD_450_ of HI-NHS-treated SARS-CoV-2)/(OD_450_ of Triton X-100-treated SARS-CoV-2 – OD_450_ of cell culture medium-treated SARS-CoV-2)] x 100*.

### Serum sensitivity TCID_50_ assay

Vero76-ACE2 cells were seeded in complete 10% FCS DMEM at 1 x 10^4^ cells per well in flat-bottom 96-well plates (Greiner) and incubated at 37°C and 5% CO_2_. The next day, normalised inputs of SARS-CoV-2 (10^7^TCID_50_/ml) were treated with 50%, 20% and 10% niNHS (Dunn Labortechnik GmbH), HI-niNHS or DMEM for 1 h at 37°C. Complement-mediated virolysis was assessed by serially diluting the samples on the Vero76-ACE2 monolayer and quantifying the remaining infectivity via TCID_50_ assay.

### SARS-CoV-2 binding to soluble complement regulators

10^5^ plaque-forming units (PFU)/well of SARS-CoV-2 were coated on Nunc™ MaxiSorp™ ELISA plates (Sigma) overnight at 4°C. Plates were washed with 0.1% BSA in PBS, fixed with 4% formaldehyde (Carl Roth) and blocked with 5% dry milk in PBS, followed by incubation with 100 µl of 1:50-diluted HI-niNHS as a source of soluble complement regulators, for 1 h at 37°C. After washing, binding was detected with goat anti-human FH pAb (#341276, Sigma, 15 µg/ml), mouse anti-human clusterin mAb (#sc-166907, Santa Cruz Biotechnology, 5 µg/ml), mouse anti-human C4bp mAb (#sc-398720, Santa Cruz Biotechnology, 5 µg/ml), followed by anti-mouse IgG HRP-conjugated (#115-035-003, Jackson ImmunoResearch, diluted 1:10000 in blocking buffer) or anti-goat IgG HRP-conjugated (#305-035-045, Jackson ImmunoResearch, diluted 1:10000 in blocking buffer) secondary Abs. ELISA was developed using the TMB substrate and TMB Blue STOP solution (Sera Care) according to the manufacturer’s instructions and OD_650_ was measured on a BioRad plate reader.

### SARS-CoV-2 EM fusion protein binding to FH

Nunc™ MaxiSorp™ ELISA plates (Sigma) were coated with recombinant His-tagged SARS-CoV-2 envelope (E) membrane (M) fusion protein (EM) at 0.25 µg/well, or BSA (Carl Roth) as control overnight at 4°C. FH was provided as a purified protein (#HC2130, Hycult Biotech) or from hiNHS and added to the plates in 2-fold dilutions starting at 1 µg/well. Binding was analysed using the mouse anti-human FH mAb (Santa Cruz Biotechnology, 15 µg/ml), followed by anti-mouse IgG HRP-conjugated Abs (#115-035-003, Jackson ImmunoResearch, diluted 1:10000 in blocking buffer). ELISA was developed using the TMB substrate and TMB Blue STOP solution (Sera Care) according to the manufacturer’s instructions and OD_650_ was measured on a BioRad plate reader.

### SARS-CoV-2 binding to recombinant FH-derived SCRs

Nunc™ MaxiSorp™ ELISA plates (Sigma) were coated with 10^5^ PFU/well of SARS-CoV-2 overnight at 4°C, washed with 0.1% BSA in PBS, fixed with 4% formaldehyde (Carl Roth) and blocked with 5% dry milk in PBS. Plates were then incubated with recombinant FH-derived SCR18-20 or control SCR16-17 in the indicated concentrations for 1 h at 37°C, followed by washing and detection with goat anti-human FH mAb (Sigma, 15 µg/ml) and anti-goat IgG HRP-conjugated Abs (#305-035-045, Jackson ImmunoResearch, diluted 1:10000 in blocking buffer). ELISA was developed using the TMB substrate and TMB Blue STOP solution (Sera Care) according to the manufacturer’s instructions and OD_650_ was measured on a BioRad plate reader.

### Analysis of SARS-CoV-2 S- and RBD-specific RBD IgG Abs

Nunc™ MaxiSorp™ ELISA plates (BioLegend) were coated with 1 µg/ml recombinant His-tagged SARS-CoV-2 S (S1+S2) protein (ancestral strain; kindly provided by Renate Kunert and Patrick Mayrhofer, Institute of Animal Cell Technology and Systems Biology, BOKU, Vienna) or recombinant His-tagged SARS-CoV-2 RBD protein (ancestral strain; #Z03483, GenScript) overnight at 4°C. Plates were washed three times with PBS+0.05% Tween (PBST), blocked with 10% FCS+3% BSA in PBST and incubated with 100 µl HI-niNHS or HI-iNHS samples (diluted 1:1000 in 3% BSA in PBST) for 1 h at RT. Following three washing steps, samples were incubated with mouse anti-human IgG Fc HRP-conjugated mAb (#A01854, GenScript, 1:10000 in blocking buffer) and serum Ab levels were determined by ELISA, using the DY999 substrate solution (R&D Systems) according to the manufacturer’s instructions. OD_450_ was measured with a Tecan Spark® Multimode Microplate Reader (Tecan). Background absorption (PBS-coated wells) for each serum was subtracted from the corresponding OD_450_ values.

### Live virus neutralisation assay

Vero cells were seeded in complete 10% FCS DMEM at 1 x 10^4^ cells per well in flat-bottom 96-well plates (Greiner) and incubated at 37°C and 5% CO_2_. The next day, 800 TCID_50_ of SARS-CoV-2 isolate BetaCoV/Germany/BavPat1/2020 (ancestral strain) were mixed with 3-fold dilutions of the indicated HI-iNHS in 2% FCS DMEM. After incubation for 1 h at RT, the virus-serum mixture was added to Vero cell monolayers at 600 TCID_50_/well and incubated for 48 h at 37°C and 5% CO_2_. Cells were then sequentially fixed with 10% formalin in PBS for 10 min and 5% formalin in PBS for 20 min, washed with PBS and permeabilised with 0.1% Triton X-100 for 15 min at RT. After blocking with 10% FCS in PBS+0.05% Tween (PBST), cells were stained by indirect immunofluorescence using rabbit anti-SARS-CoV-2 N mAb (#40143-R019, SinoBiological, 1:15000 in blocking buffer), followed by HPR-conjugated goat anti-rabbit Ab (#1706515, BioRad, 1:10000 in blocking buffer). The in-cell ELISA was developed with the DY999 substrate solution (R&D Systems) according to the manufacturer’s instructions and OD_450_ (and background correction at OD_630_) was measured with a Tecan Spark® Multimode Microplate Reader (Tecan). The neutralisation capacity of each serum sample is expressed as the % inhibition of infection for each well and was calculated with the following formula: *100 – [(X - average of ʻno virusʼ wells)/(average of ʻvirus onlyʼ wells - average of ʻno virusʼ wells)*100],* where X is the background-subtracted read for each well.

### Flow cytometry

Flow cytometry analysis was performed as previously described [12]. Briefly, Jurkat E6.1 cells were washed with cold staining buffer (PBS + 1% BSA and 0.02% NaN_3_), blocked with 4.8 mg/ml human IgG (Beriglobin P, CSL Behring) for 30 min on ice and incubated with FITC-conjugated CD59 MEM-43 mAb (EXBIO, 1:100), CD48 MEM-102 mAb (kindly provided by Vaclav Horejsi, 10 µg/ml) or staining buffer for 30 min on ice. Cells were washed twice with staining buffer, followed by incubation with AF555-labelled CD59 MEM-43 Fab (10 µg/ml) or AF555-labelled CD48 MEM-102 Fab (10 µg/ml) either alone or as a secondary step for CD59 MEM-43 and CD48 MEM-102 mAbs respectively, or FITC-conjugated anti-mouse IgM + IgG F(ab’)_2_ (An der Grub Bio Research GmbH, 1:300) as a secondary step for CD48 MEM-102 mAb, for 30 min on ice. Samples were again gently washed twice and treated with DAPI (Sigma, 0.5 µg/ml) for the exclusion of dead cells and recorded on a Fortessa flow cytometer (BD Biosciences). Further data analysis was performed using the FlowJo software (BD Biosciences).

### Statistical analysis

Calculations were performed using Excel 365 (Microsoft). Figures and statistical analyses were produced with GraphPad Prism 7. Statistical significance between different groups was calculated using the tests indicated in each figure legend.

**Table S2. Resources and reagents used in the study.**

| **REAGENT or RESOURCE** | **SOURCE** | **IDENTIFIER** |
| --- | --- | --- |
| **Abs** | | |
| SARS-CoV-2 Spike (S1) mouse mAb | SinoBiological | Cat# 40591-MM42 |
| SARS-CoV-2 Spike (S1) human mAb (clone AM001414) | BioLegend | Cat# 938701 |
| SARS-CoV-2 Nucleocapsid (N) mouse mAb | SinoBiological | Cat# 40143-MM05 |
| SARS-CoV-2 Nucleocapsid (N) rabbit mAb | SinoBiological | Cat# 40143-R019 |
| Purified anti-SARS-CoV-2 Nucleocapsid (N) mAb (clone A20087F) | BioLegend | Cat# 940902 |
| CD55/DAF rabbit mAb | Cell Signaling | Cat# 31759 |
| CD59 rabbit mAb | Cell Signaling | Cat# 25393 |
| Actin rabbit pAb | Sigma-Aldrich | Cat# A2066 |
| CD46 rabbit mAb | Cell Signaling | Cat# 13241 |
| CD14 mouse mAb (clone MEM-18) | Laboratory of Vaclav Horejsi, Institute of Molecular Genetics, Academy of Sciences of the Czech Republic, Prague, Czech Republic | N/A |
| CD59 mouse mAb (clone MEM-43) | Laboratory of Vaclav Horejsi, Institute of Molecular Genetics, Academy of Sciences of the Czech Republic, Prague, Czech Republic | N/A |
| CD59 mouse mAb (clone MEM-43/5) | Laboratory of Vaclav Horejsi, Institute of Molecular Genetics, Academy of Sciences of the Czech Republic, Prague, Czech Republic | N/A |
| CD55 mouse mAb (clone BRIC-216) | Santa Cruz Biotechnology | Cat# sc-59092 |
| CD59 mouse mAb (clone MEM-43) FITC conjugate | EXBIO | Cat# 1F-233-T100 |
| CD48 mouse mAb (clone MEM-102) | Laboratory of Vaclav Horejsi, Institute of Molecular Genetics, Academy of Sciences of the Czech Republic, Prague, Czech Republic | N/A |
| CD59 Fab (clone MEM-43) labelled with Alexa Fluor (AF)555 | Produced in house, as described in [11] | Wieser et al. [11]  Drbal et al. [10] |
| CD48 Fab (clone MEM-102) labelled with AF555 | Produced in house, as described in [11] | Wieser et al. [11]  Drbal et al. [10] |
| Purified mouse IgG2a, κ isotype control mAb (clone MOPC-173) | BioLegend | Cat# 400264 |
| Purified mouse IgG1, κ isotype control mAb (clone MOPC-21) | BioLegend | Cat# 400101 |
| Purified human IgG1, κ isotype control mAb (clone QA16A12) | BioLegend | Cat#403501 |
| Mouse IgG1 isotype control mAb (clone PPV06) | Laboratory of Vaclav Horejsi, Institute of Molecular Genetics, Academy of Sciences of the Czech Republic, Prague, Czech Republic | N/A |
| Mouse IgG2b isotype control mAb (PPV02) | Laboratory of Vaclav Horejsi, Institute of Molecular Genetics, Academy of Sciences of the Czech Republic, Prague, Czech Republic | N/A |
| Factor H goat pAb | Sigma-Aldrich | Cat# 341276 |
| Factor H mouse mAb | Santa Cruz Biotechnology | N/A |
| Clusterin mouse mAb | Santa Cruz Biotechnology | Cat# sc-166907 |
| C4bp mouse mAb | Santa Cruz Biotechnology | Cat# 398720 |
| Goat anti-rabbit IgG-HRP conjugate | BioRad | Cat# 1706515 |
| Rabbit anti-mouse IgG-HRP conjugate | Sigma-Aldrich | Cat# A9044 |
| Rabbit anti-goat IgG-HRP conjugate | Jackson ImmunoResearch | Cat# 305-035-045 |
| Goat anti-mouse IgG-HRP conjugate | Jackson ImmunoResearch | Cat# 115-035-003 |
| Mouse anti-human IgG Fc-HRP conjugate | GenScript | Cat# A01854 |
| Anti-mouse IgM + IgG F(ab’)_2_ FITC conjugate | An der Grub Bio Research GmbH | N.A. |
| **Bacterial and virus strains** | | |
| SARS-CoV-2 isolate BetaCoV/Germany/BavPat1/2020 (ancestral strain) | Laboratory of Christian Drosten (Charité, Berlin, Germany) | Ref-SKU: 026V-03883 |
| SARS-CoV-2 isolated at the Medical University of Innsbruck in 2020 (isolate 1.2, ancestral strain) | Laboratory of Janine Kimpel, Institute of Virology, Medical University of Innsbruck | N/A |
| **Biological samples** |  |  |
| NHS | Dunn Labortechnik GmbH | N/A |
| NHS from SARS-CoV-2-vaccinated healthy donors, obtained at the Medical University of Vienna | Medical University of Vienna, Vienna, Austria | N/A |
| **Chemicals, peptides, and recombinant proteins** | | |
| Capto Core 700 multimodal chromatography resin | Cytiva | Cat# |
| Phosphatidylinositol-specific phospholipase C (PI-PLC), from Bacillus cereus | Sigma-Aldrich | Cat# P5542-25 |
| Recombinant Factor H-derived SCR18-20 | Group of Heribert Stoiber, Institute of Virology, Medical University of Innsbruck | Hörl et al. [13]  Prantl et al. [14] |
| Recombinant Factor H-derived SCR16-17 | Group of Heribert Stoiber, Institute of Virology, Medical University of Innsbruck | Hörl et al. [13]  Prantl et al. [14] |
| Recombinant Factor H-derived SCR11-12 | Group of Heribert Stoiber, Institute of Virology, Medical University of Innsbruck | Hörl et al. [13]  Prantl et al. [14] |
| Factor H | Hycult Biotech | Cat# HC2130 |
| Recombinant SARS-CoV-2 Spike (S1+S2) protein, His-tagged | Patrick Mayrhofer and Renate Kunert, Institute of Animal Cell Technology and Systems Biology, BOKU University, Vienna | Mayrhofer et al. [15] |
| Recombinant SARS-CoV-2 receptor binding domain (RBD) protein, His-tagged | GenScript | Cat# Z03483 |
| SARS-CoV-2 envelope (E) membrane (M) fusion protein (EM), His-tagged | Native Antigen Company | N/A |
| **Critical commercial assays** | | |
| **Deposited data** | | |
| LC-MS/MS data <https://www.ebi.ac.uk/pride/> | This paper | PRIDE identifier: PXD050009 |
| **Experimental models: Cell lines** | | |
| Caco-2 cells | ATCC | HTB37 (ATCC) |
| Vero cells | Laboratory of Sylvia Knapp, Department of Medicine I, Medical University Vienna, Vienna, Austria | CCL-81 (ATCC) |
| Vero76 cells | Laboratory of Janine Kimpel, Institute of Virology, Medical University of Innsbruck | N/A |
| Vero76-ACE2 cells | Laboratory of Janine Kimpel, Institute of Virology, Medical University of Innsbruck | N/A |
| Jurkat E6.1 cells | ATCC | TIB-152 (ATCC) |
| **Experimental models: Organisms/strains** | | |
| **Oligonucleotides** | | |
| **Recombinant DNA** | | |
| **Software and algorithms** | | |
| GraphPad Prism version 7.0 | GraphPad Software | <https://www.graphpad.com> |
| Fiji Image Analysis Software | ImageJ | <https://imagej.net/software/fiji/> |
| Excel365 | Microsoft | <https://www.microsoft.com/de-at/microsoft-365/excel> |
| Proteome Discoverer 2.4 | Thermo Fisher | <https://www.thermofisher.com/> |
| R 4.3.3 | R Core Team (2023) | <https://www.r-project.org/> |
| FlowJo | BD Biosciences | <https://www>.flowjo.com/solutions/flowjo |
| **Other** | | |
| Dulbecco’s Modified Eagle’s Medium (DMEM), high glucose, GlutaMAX, pyruvate | Gibco | Cat# 10569010 |

**
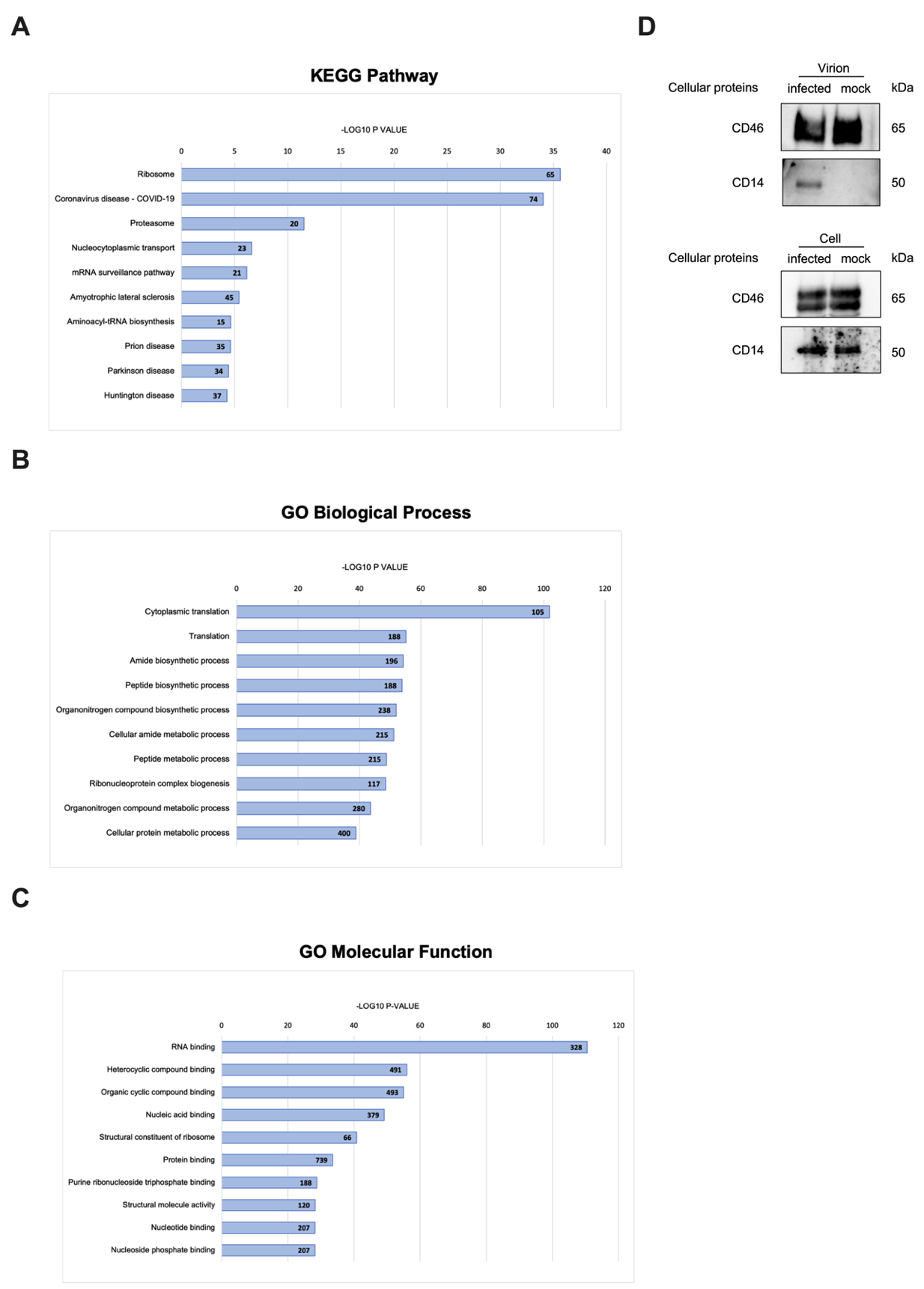
**

**Figure S1. Functional characterisation of SARS-CoV-2-associated cellular proteins.**

(A-C) KEGG Pathway (A) and GO Biological Process (B) and Molecular Function (C) gene enrichment analyses of the 808 statistically enriched cellular proteins identified in the purified SARS-CoV-2 preparations from Figure 1D. Enrichment is plotted as a function of
-log10 p value and represented by the length of the blue bars. The number of proteins (counts) associated with each term is depicted in bold in the corresponding bar. Top ten categories are shown. (D) Immunoblot analysis of host cellular CD46 and CD14 in purified SARS-CoV-2 and mock preparations (top) and Caco-2 cell lysates (bottom).


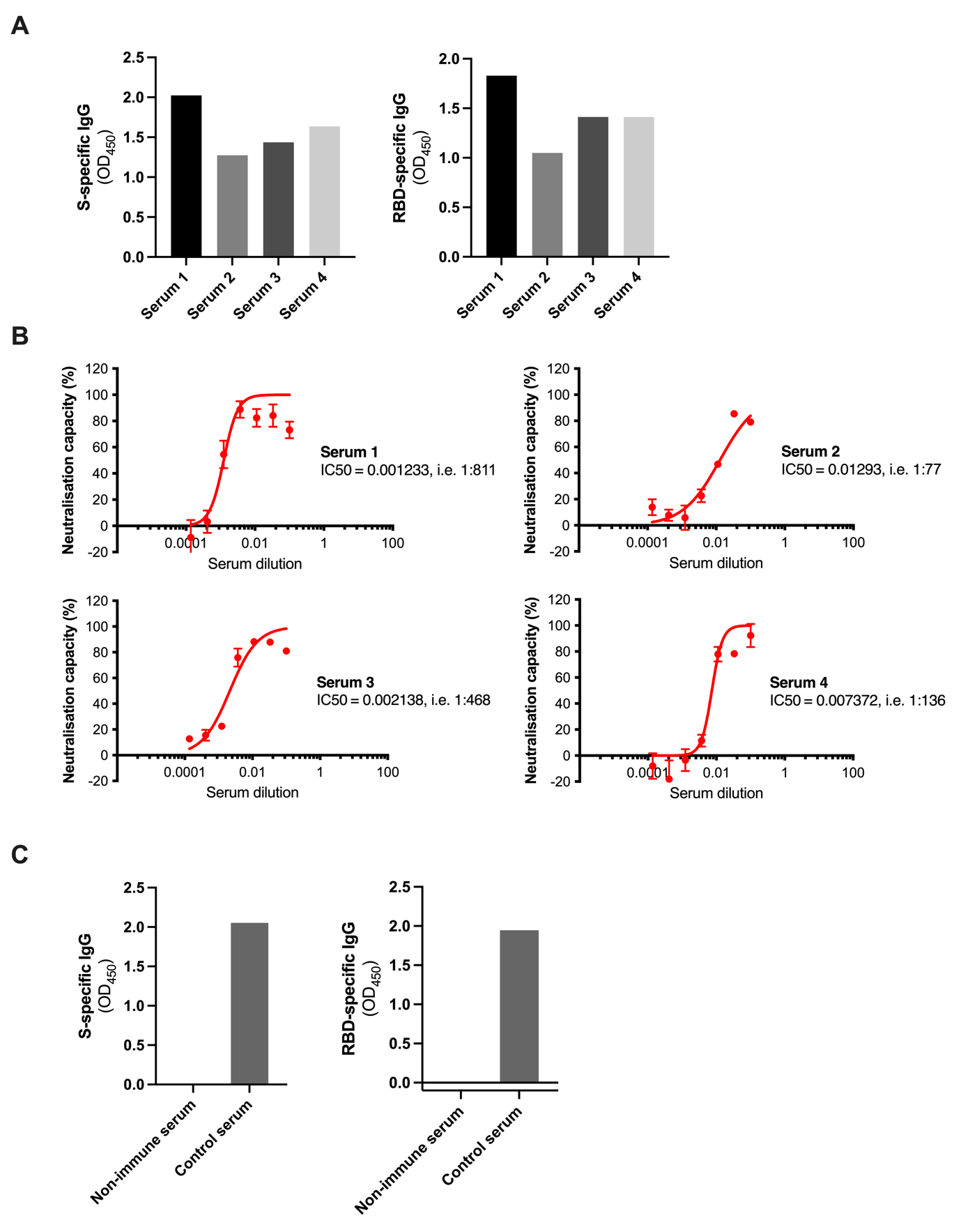


**Figure S2. Levels of SARS-CoV-2-specific Abs in the used human sera.**

(A) Analysis of total SARS-CoV-2 (ancestral strain) S- and RBD-specific IgG Abs in the utilised iNHS by ELISA. (B) Neutralisation capacities (expressed as % inhibition of infection) of the used iNHS against the SARS-CoV-2 ancestral strain, determined by live virus neutralisation assay. (C) Analysis of total SARS-CoV-2 (ancestral strain) S- and RBD-specific IgG Abs in the utilised non-immune serum and an immunised serum used as positive control (control serum).


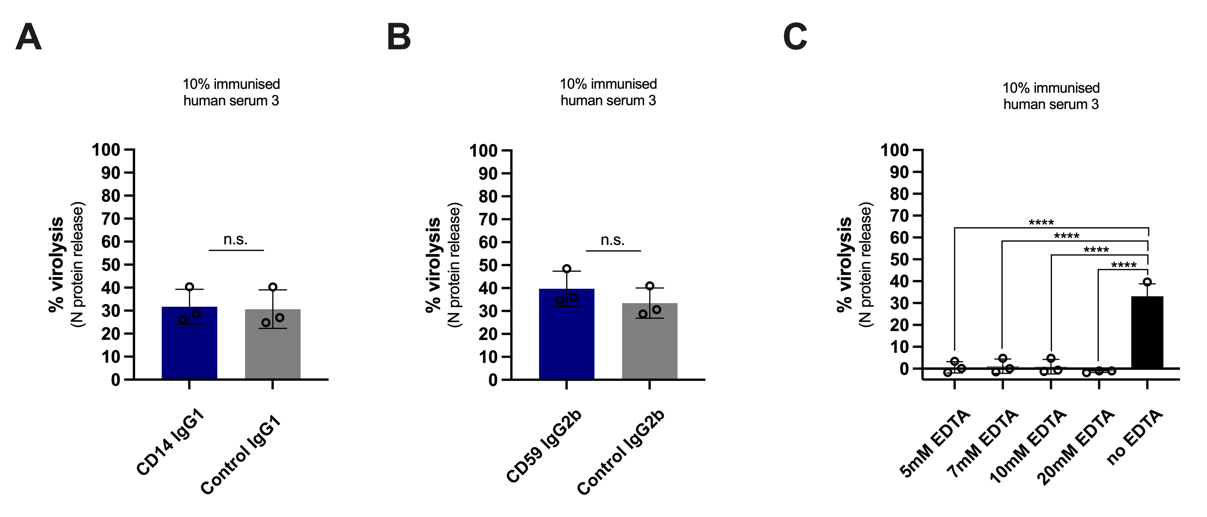


**Figure S3. Non-blocking mAbs do not enhance complement-mediated virolysis and complement inactivation through ion chelation completely abolishes virolysis.**

(A, B) ADCML of SARS-CoV-2 incubated with CD14 blocking mAb (A) or CD59 non-blocking mAb (clone MEM-43/5) (B), or the corresponding isotype control mAbs. Virolysis is expressed as % of Triton X-100-treated virions. Data are represented as mean ± SD (*n* = 3 independent experiments), and statistical significance was assessed by two-tailed unpaired Student’s *t*-test. (C) Abrogation of ADCML of SARS-CoV-2 by EDTA treatment. Virolysis is expressed as % of detergent Triton X-100-treated virions. Data are represented as mean ± SD (*n* = 3 independent experiments), and statistical significance was evaluated by one-way ANOVA with Dunnett’s multiple comparisons test; *****p < 0.0001*.


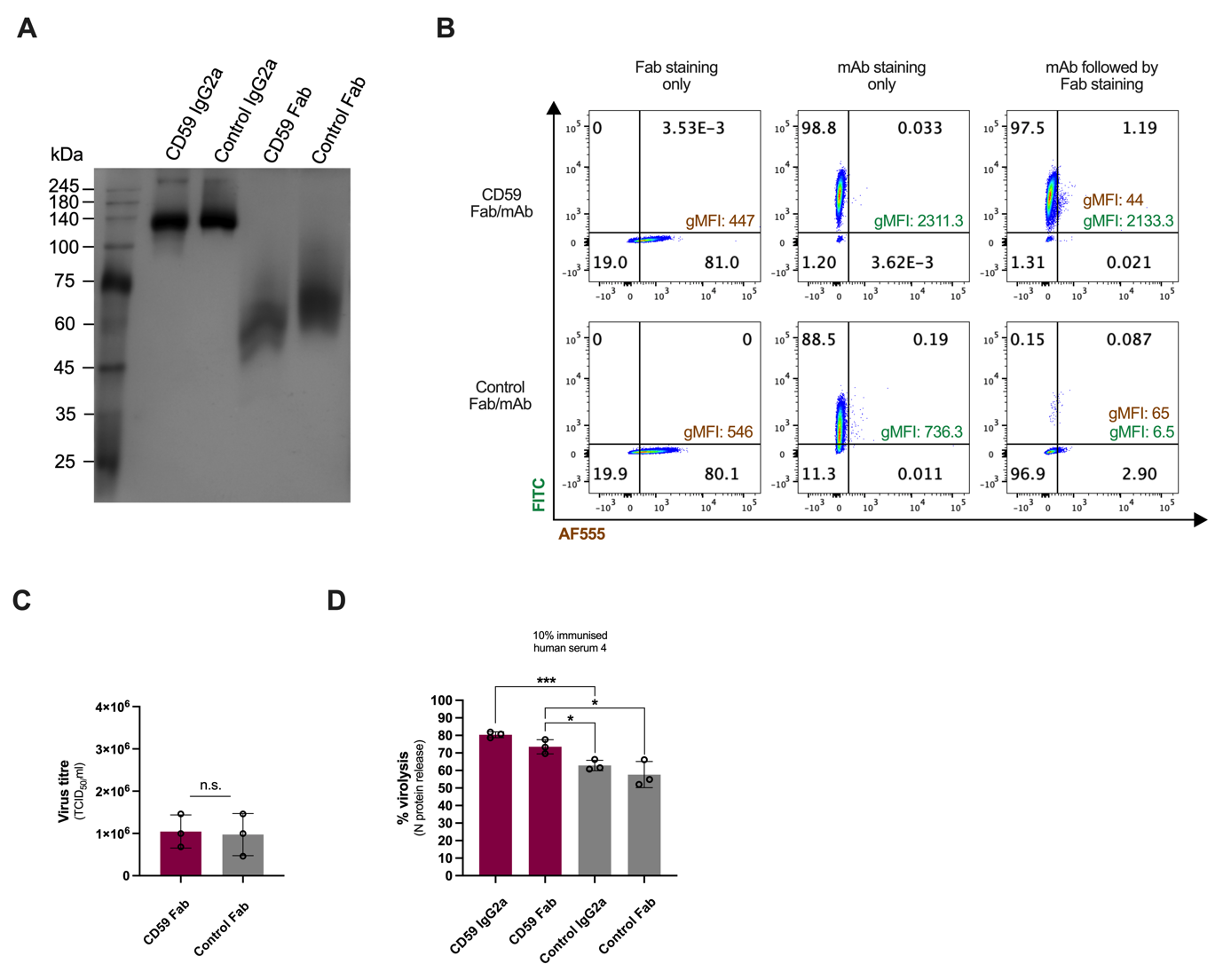


**Figure S4. Structural and functional characterisation of CD59 and control Fab fragments.**

(A) Non-reducing 10% SDS-PAGE and silver staining analysis of CD59 mAb MEM-43, isotype control mAb, CD59 Fab MEM-43 and control Fab (CD48 MEM-102). (B) Flow cytometry analysis of CD59 Fab MEM-43 and control Fab. Jurkat E6.1 cells were incubated with AF555-labelled CD59 Fab or AF555-labelled control (CD48) Fab alone (left column), FITC-conjugated CD59 mAb alone or non-labelled CD48 mAb followed by FITC-conjugated anti-mouse IgM + IgG F(ab’)_2_ (middle column), or FITC-conjugated CD59 mAb followed by AF555-labelled CD59 Fab or non-labelled CD48 mAb followed by AF555-labelled control (CD48) Fab (right column). CD59 Fab and/or mAb staining is shown in the top row and control CD48 Fab and/or mAb staining in the bottom row. Signals are also expressed as geometric mean fluorescence intensity (gMFI) corrected for the background autofluorescence of non-stained cells. One representative staining of four independent experiments is shown. (C) Titres of SARS-CoV-2 incubated with CD59 Fab or control Fab, determined by TCID_50_ assay. Data are represented as mean ± SD (*n* = 3 independent experiments) and statistical significance was evaluated by two-tailed unpaired Student’s *t*-test. (D) Enhancement of ADCML of SARS-CoV-2 by CD59 blocking mAb, CD59 Fab, or isotype control mAb and control Fab. Virolysis is expressed as % of Triton X-100-treated virions. Data are represented as mean ± SD (*n* = 3 independent experiments) and statistical significance was assessed by two-tailed unpaired Student’s *t*-test; **p* *<* *0.05, ***p* *<* *0.001.*

**Supplemental references**

[1] James KT, Cooney B, Agopsowicz K, et al. Novel High-throughput Approach for Purification of Infectious Virions. Scientific Reports. 2016 2016/11/09;6(1):36826.

[2] Ramakrishnan MA. Determination of 50% endpoint titer using a simple formula. World J Virol. 2016 May 12;5(2):85-6.

[3] Chevallet M, Luche S, Rabilloud T. Silver staining of proteins in polyacrylamide gels. Nat Protoc. 2006;1(4):1852-8.

[4] Hughes CS, Foehr S, Garfield DA, et al. Ultrasensitive proteome analysis using paramagnetic bead technology. Mol Syst Biol. 2014 Oct 30;10(10):757.

[5] Rappsilber J, Mann M, Ishihama Y. Protocol for micro-purification, enrichment, pre-fractionation and storage of peptides for proteomics using StageTips. Nat Protoc. 2007;2(8):1896-906.

[6] Olsen JV, de Godoy LM, Li G, et al. Parts per million mass accuracy on an Orbitrap mass spectrometer via lock mass injection into a C-trap. Mol Cell Proteomics. 2005 Dec;4(12):2010-21.

[7] Perez-Riverol Y, Csordas A, Bai J, et al. The PRIDE database and related tools and resources in 2019: improving support for quantification data. Nucleic Acids Res. 2019 Jan 8;47(D1):D442-d450.

[8] Sherman BT, Hao M, Qiu J, et al. DAVID: a web server for functional enrichment analysis and functional annotation of gene lists (2021 update). Nucleic Acids Res. 2022 Jul 5;50(W1):W216-w221.

[9] Huang da W, Sherman BT, Lempicki RA. Systematic and integrative analysis of large gene lists using DAVID bioinformatics resources. Nat Protoc. 2009;4(1):44-57.

[10] Drbal K, Moertelmaier M, Holzhauser C, et al. Single-molecule microscopy reveals heterogeneous dynamics of lipid raft components upon TCR engagement. Int Immunol. 2007 May;19(5):675-84.

[11] Wieser S, Moertelmaier M, Fuertbauer E, et al. (Un)confined diffusion of CD59 in the plasma membrane determined by high-resolution single molecule microscopy. Biophys J. 2007 May 15;92(10):3719-28.

[12] Ohradanova-Repic A, Machacek C, Charvet C, et al. Extracellular Purine Metabolism Is the Switchboard of Immunosuppressive Macrophages and a Novel Target to Treat Diseases With Macrophage Imbalances. Front Immunol. 2018;9:852.

[13] Hörl S, Bánki Z, Huber G, et al. Reduction of complement factor H binding to CLL cells improves the induction of rituximab-mediated complement-dependent cytotoxicity. Leukemia. 2013 Nov;27(11):2200-8.

[14] Prantl L, Heider P, Bergmeister L, et al. Enhancement of complement-dependent cytotoxicity by linking factor-H derived short consensus repeats 19-20 to CD20 antibodies. Front Immunol. 2024;15:1379023.

[15] Mayrhofer P, Hunjadi M, Kunert R. Functional Trimeric SARS-CoV-2 Envelope Protein Expressed in Stable CHO Cells. Front Bioeng Biotechnol. 2021;9:779359.
